# Zerumbone ameliorates the inflammatory response and organ damage in severe acute pancreatitis via the ROS/NF-κB pathway

**DOI:** 10.1186/s12876-023-02962-6

**Published:** 2023-09-27

**Authors:** Fengmei Zhang, Dongjia Xu

**Affiliations:** Department of Gastroenterology, Haining People’s Hospital, Jiaxing City, 314400 Zhejiang China

**Keywords:** Zerumbone, Severe acute pancreatitis, Organ damage, ROS, NF-κB

## Abstract

**Objective:**

The aim of the current study was to determine the mechanism by which Zerumbone (ZER) ameliorates inflammation and organ damage in a rat model of severe acute pancreatitis (SAP).

**Methods:**

Different concentrations of ZER (10, 20 and 40 mg/kg) were administered by femoral vein puncture 30 min prior to establishment of the SAP model. Hematoxylin and eosin (H&E) staining was used to assess pathological changes in the pancreatic tissue of SAP-induced rats. The lung wet/dry (W/D) ratio was assessed and serum levels of amylase (AMY), alanine aminotransferase (ALT), creatinine (Cr), aspartate aminotransferase (AST) and phospholipase A_2_ (PLA_2_) were measured. Western blot analysis was used to examine changes in the expression of ROS/NF-κB pathway-associated proteins.

**Results:**

SAP was confirmed by significant histopathological damage to the pancreas. ZER (10, 20 and 40 mg/kg) was found to alleviate pancreatitis and decrease ascites volume, lung W/D ratio, pancreatic pathology score, oxidative stress and inflammatory damage. High concentrations (20 and 40 mg/kg) of ZER were shown to increase levels of hepatorenal toxicity. In contrast, 10 mg/kg ZER was found to attenuate liver enzyme levels, reduce pathological damage to the liver, and protect against extrapancreatic organ damage to the liver in SAP-induced rats. Moreover, ZER showed no significant side effects in normal rats. Finally, we demonstrated that ZER mediated its anti-inflammatory effects on SAP through the ROS/NF-κB signaling pathway.

**Conclusion:**

ZER alleviated SAP-induced oxidative stress and inflammatory injury via the ROS/NF-κB pathway, and had a protective effect on lung injury and liver damage.

**Supplementary Information:**

The online version contains supplementary material available at 10.1186/s12876-023-02962-6.

## Introduction

Acute pancreatitis (AP) is an inflammatory disease that is characterized by the activation of pancreatic enzymes, which leads to the digestion, edema, hemorrhage and even necrosis of pancreatic tissue. Clinical manifestations of SP include acute abdominal pain, abdominal distension, nausea, vomiting, fever and elevated blood pancreatic enzymes. In particular, severe acute pancreatitis (SAP) is diagnosed when patients with AP develop multiple complications including pancreatic hemorrhage and necrosis, secondary infection, peritonitis and shock with persistent organ failure (> 48 h). Statistical evidence indicates that AP progresses to SAP in 10–20% of patients [1–5]. Due to the rapid onset and progression of the disease, SAP is associated with a relatively high mortality rate and overall poor prognosis.

Although inflammatory responses, oxidative stress injury, apoptosis and microcirculatory disorders have been shown to contribute to the activation of trypsin, the pathogenesis of SAP remains unclear. Recent studies have shown that activation of pancreatic enzymes may also lead to excessive secretion of inflammatory factors and the necrosis of pancreatic tissues, thereby contributing to the development of complications including liver and lung damage [6, 7]. Therefore, in clinical practice, a treatment regimen for SAP involving fasting and early enteral nutrition was designed to prevent further deterioration of symptoms. However, this treatment did not significantly improve the overall condition of SAP patients [8, 9]. Several studies have shown that combining traditional Chinese medicine with the above treatment regimen shortens the course of the disease, reduces the possibility of developing complications and improves the overall prognosis of patients, by protecting the intestinal mucosa and reducing the systemic inflammatory response of the patients [10].

ZER is the main active ingredient in the extract of *Zingiber zerumbet (L.) smith* (a traditional Chinese medicine), and has been reported to possess anti-inflammatory and oncological properties, which are mediated through inhibition of the PI3K-mTOR, MAPK-ERK, NF-κB and NF-κB/TLR signaling pathways [11–15].

Although previous studies have reported that ZER can alleviate the damage caused by SAP [16], the underlying mechanism remains unknown. Since the ROS/NF-κB pathway may be involved in the development of various diseases including immune system disorders, cardiovascular diseases and inflammatory diseases [17–21], we hypothesized that ZER may exert its anti-inflammatory effects through the ROS/NF-κB pathway. Therefore, in the present study, we examined whether ZER alleviated histopathological damage, inflammation and oxidative stress levels, and adjacent organ damage in SAP-induced rats through the ROS/NF-κB pathway in order to provide a reference for the clinical prevention and treatment of SAP.

## Materials and methods

### Animals

Thirty-six male SPF-grade Wistar rats, weighing 200 − 250 g and aged 8–10 weeks, were purchased from Beijing baiaosike Biomedical Technology Co., Ltd. All rats were acclimatized and kept for 7 days at a room temperature of 22 − 24 °C and relative humidity of 50−60% in animal housing with free access to standard chow and sterile distilled water. The study was approved by the Experimental Animal Ethics Committee of Beijing baiaosike Biomedical Technology Co., Ltd ethics committee (No. MDL 2023-02-11-01).

### Establishment of the SAP model

Prior to construction of the SAP model, rats had unrestricted access to food and water. Rats were then weighed and randomly assigned to groups. Rats were anaesthetized by intraperitoneal injection of 10% chloric acid hydrate (0.3 mL/100 g; PM12563, Perfemiker, Shanghai, China) solution. Once anaesthetized, the limbs and incisors were fixed with a rubber band, and the animals were placed onto the operating table, where their skin was prepared and disinfected. After covering the rats with a sterile towel sheet, a median incision was made into the upper abdomen to expose the duodenum and pancreaticobiliary duct. The pancreaticobiliary duct was double clamped near the hilar with a noninvasive vascular clamp, and the duodenum was pierced with a scalp needle (diameter of 0.4 mm). A retrograde puncture of the cholangiopancreatic duct was then performed through the duodenal papilla. The needle was fixed in position with a noninvasive vascular clamp, and freshly prepared 5% sodium taurocholate solution (0.1 mL/100 g; T0875, Sigma-Aldrich, USA) was injected into the biliopancreatic duct at a constant speed (0.1 mL/min). Next, the scalp needle was removed and the vascular clamp remained in position for a further 5 min to allow the sodium taurocholate solution to fully enter each pancreatic lobule. After 5 min, the vascular clamp was loosened. Congestion and edema of the pancreatic tissue, as well as the duodenum, were observed by the naked eye. Then, the abdomen was closed in layers. Finally, rats were injected subcutaneously with normal saline.

### Animal grouping and drug intervention

Thirty-six SPF male rats were randomly assigned to the following six groups (n = 6): normal control group (control), SAP group, ZER (471-05-6, Kingherbs, Changsha, China) treatment group (10, 20, 40 mg/kg) group and ZER drug control group (40 mg/kg ZER). A rat model of SAP was established as described earlier. The control group was treated the same as the SAP group, except rats were injected into the biliopancreatic duct with normal saline instead of 5% sodium taurocholate solution. The ZER control group was treated the same as the normal control group, except 40 mg/kg of ZER was administered by femoral vein puncture 30 min prior to saline injection. The ZER treatment groups were administered different concentrations of ZER by femoral vein puncture 30 min prior to induction of SAP.

### Sample collection

Twelve hours post-SAP, blood (approximately 3 mL) was drawn by cardiac puncture with a 5 mL syringe, centrifuged at 3000 rpm/min for 15 min in a high speed centrifuge, and the supernatant was collected and stored at -20 °C in EP tubes. Rat ascites was absorbed with dry cotton balls. Pancreatic tissue was removed from rats in each of the treatment groups, fixed with 4% paraformaldehyde for 24 h, dehydrated in gradient alcohol, soaked in xylene and embedded in paraffin. The lung wet-to-dry (W/D) ratio was measured using the middle lobe of the right lung of the rats. The formula for calculating the amount of ascites was the weight of wet cotton ball - weight of dry cotton ball [22].

### Hematoxylin and eosin (H&E) staining

After treatment, rats were humanely killed by cervical dislocation, and pancreatic tissue was quickly isolated, fixed in 10% neutral formaldehyde solution, paraffin-embedded, sectioned and stained with H&E. Histopathological damage to the pancreas was then visualized and photographed.

### Pulmonary W/D ratio

The middle lobe of the right lung was isolated. Blood and water were cleaned from the surface of the lung tissue with absorbent paper. The lung tissue was wrapped in aluminum foil (3 × 3 cm^2^ square) and weighed on an electronic scale (wet lung weight). Then, the lung tissue was placed in an oven at 70 °C for 24 h and reweighed to give a value for the dry weight. The W/D ratio was calculated using the following formula: W/D ratio = wet weight of lung - weight of tinfoil / dry weight of lung - weight of aluminum foil.

### Measurement of pancreatic biochemical parameters

Pancreatic tissue stored in liquid nitrogen was added to prechilled saline (4^o^C) at a ratio of 1:9, homogenized, and the supernatant was collected, divided and frozen. SOD, MDA, NO, GSH and GST levels in the pancreatic tissue were determined spectrophotometrically according to the instructions of the corresponding kits. The kits were purchased from Wuhan Saipei Biotechnology Co., Ltd (Wuhan, China) and are as follows: SP12914 for SOD, SP30131 for MDA, SP13028 for NO, SP12673 GSH, and SP12891 for GST.

### ELISA

Following treatment, 4 mL of blood was drawn from the abdominal aorta of the rats, centrifuged at 3000 rpm for 10 min and the supernatant was collected. Levels of serum amylase (AMY), alanine aminotransferase (ALT), creatinine (Cr), aspartate aminotransferase (AST), phospholipase A_2_ (PLA_2_), TNF-α, IL-1β, IL-6, IL-4 and IL-10 were measured using the corresponding ELISA kits according to the manufacturer’s instructions. The ELISA kits were purchased from Shanghai Zhen Ke Biological Technology Co., Ltd. (Shanghai, China) and are as follows: ZK-6864 for AMY, ZK-6809 for ALT, ZK-7219 for AST, ZK-6963 for Cr, ZK-6363 for PLA_2_, ZK-6601 for TNF-α, ZK-5809 for IL-1β, ZK-5835 for IL-6, ZK-5838 for IL-4, and ZK-5858 for IL-10. Briefly, the following step was necessary to obtain the levels of indicators measured above. Prepared diluted antibody was incubated with coating buffer overnight at 4℃. After determining the number of experimental wells, standards, samples with buffer were added to each well (depending on the types of wells). Antibodies were added and incubated at room temperature, experimental plates were washed and pre-diluted enzymes were added and incubated. Color development was carried out using TMB and the reaction was terminated by adding stop solution. Finally, OD values were read and analyzed using Curve Expert.

### Western blot analysis

Total protein was extracted from pancreatic tissue using lysis buffer. Protein samples were separated by SDS-PAGE, then transferred to PVDF membranes by the wet transfer method. Membranes were incubated in 5% skimmed milk powder at 37^º^C for 2 h. Next, membranes were incubated with antibodies against p65 (1:1,000; ab76302, Abcam, Cambridge, UK), p-p65 (1:1,000; ab43041, Abcam), I-κBα (1:1,000; ABP55384, AmyJet Scientific, Wuhan, China), p-I-κBα (1:1,000; HK5704, Shanghai Tusi Medical Technology Co., Shanghai, China), Keap1 (1:1,000; ab218815, Abcam), Nrf2 (1:1,000; ab31163, Abcam) and GAPDH (1:2,000; ab8245, Abcam) at 4 °C overnight. The next day, membranes were incubated with HRP-labelled secondary antibodies (1:2,500) at room temperature for 1 h. Protein bands were analyzed by ImageJ and quantified relative to GAPDH.

### Immunofluorescence staining

Paraffin tissue Sect. (4 μm) were dewaxed. Antigen retrieval was performed using 0.01 mol/L sodium citrate. Then, endogenous peroxidase was eliminated from the tissue by dropwise addition of 3% catalase. Next, samples were incubated with 5% BSA for 1 h, then incubated overnight at 4 °C with a primary antibody against Keap1 (1:100 dilution; added dropwise).

### Statistical analysis

Data were analyzed using SPSS 20.0 statistical software. Statistical data are presented as the average ± SD. One-way ANOVA was used to compare groups, while LSD was used for two-way comparisons. Then, α = 0.05 was considered to be statistically significant.

## Results

### ZER pretreatment alleviates pathological tissue damage in SAP-induced rats

**Fig. 1:**
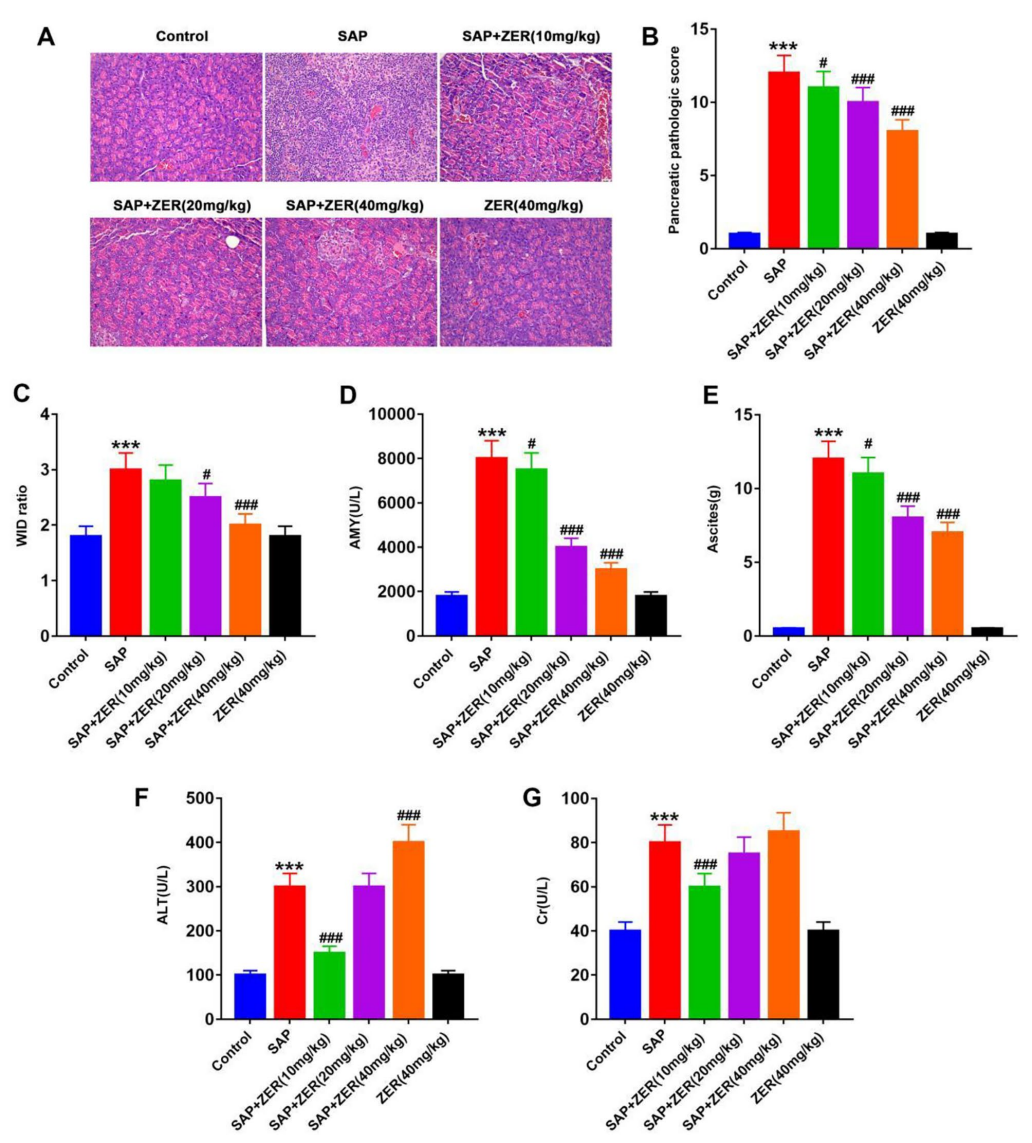
Pretreatment with ZER alleviates pathological tissue damage in SAP-induced rats. **(A)** Histopathological changes in the rat pancreas of each treatment group were examined by H&E staining (×200). **(B)** Histological score of pancreatic pathology in each group of rats. **(C)** Changes in the wet-to-dry ratio (W/D) of lungs in each group of rats. **(D)** Changes in serum amylase (AMY) levels in each group of rats. **(E)** Measurement of abdominal water volume in each group of rats. **(F)** Changes in serum alanine aminotransferase (ALT) levels in each group of rats. **(G)** Changes in serum creatinine (Cr) levels in rats in each treatment group. ^***^P < 0.001, compared with CON group, ^#^P < 0.05, ^###^P < 0.001, compared with the SAP group

H&E staining revealed normal pathological changes in the pancreas of control rats, with almost no edema, hemorrhage and inflammatory cell infiltration (Fig. 1A,B). In contrast, pathological changes were observed in the SAP group, including enlarged glandular cells, destruction of normal tissue structure, significant necrosis of the glandular alveoli, typical fat necrosis in some regions, significant inflammatory cell infiltration in the pancreatic lobules, significant inflammatory cell exudation from blood vessels and an increased pathological damage score (Fig. 1A,B). Notably, intravenous administration of ZER (10, 20 and 40 mg/kg) led to a reduction in pancreatic edema, alveolar necrosis, extravascular and septal hemorrhage and inflammatory cell infiltration compared to the SAP group (Fig. 1A). In addition, a concentration-dependent reduction in the pathological damage score was observed (Fig. 1B). No significant differences in pancreatic tissue pathology were observed between the drug control and control groups (P < 0.05, Fig. 1A,B). The W/D ratio of lung tissue in the SAP group was significantly higher than the control group. Treatment with ZER led to a concentration-dependent decrease in the W/D ratio of lung tissues compared to the SAP group (P < 0.05, Fig. 1C). Serum AMY levels and abdominal water volumes were significantly higher in the SAP group than the control group. ZER treatment (10, 20 and 40 mg/kg) led to a significant dose-dependent decrease in AMY and abdominal water levels compared to the SAP group. No significant differences in serum AMY levels and abdominal water volumes were found between the drug control and control groups (P < 0.05, Fig. 1D,E). In addition, significantly higher serum ALT levels were observed in the SAP group compared to the control group. Treatment with 10 mg/kg ZER led to a significant reduction in serum ALT levels compared to the SAP group, while 40 mg/kg ZER resulted in significantly higher serum ALT levels. No significant differences in serum ALT levels were observed between the ZER (20 mg/kg) and SAP groups, and drug control and control groups (P < 0.05, Fig. 1F). Serum Cr levels were significantly higher in the SAP group compared to the control group. Treatment with 10 mg/kg ZER led to a significant reduction in serum Cr levels compared to the SAP group (P < 0.05, Fig. 1G).

### ZER attenuates oxidative stress injury in SAP-induced rats

**Fig. 2:**
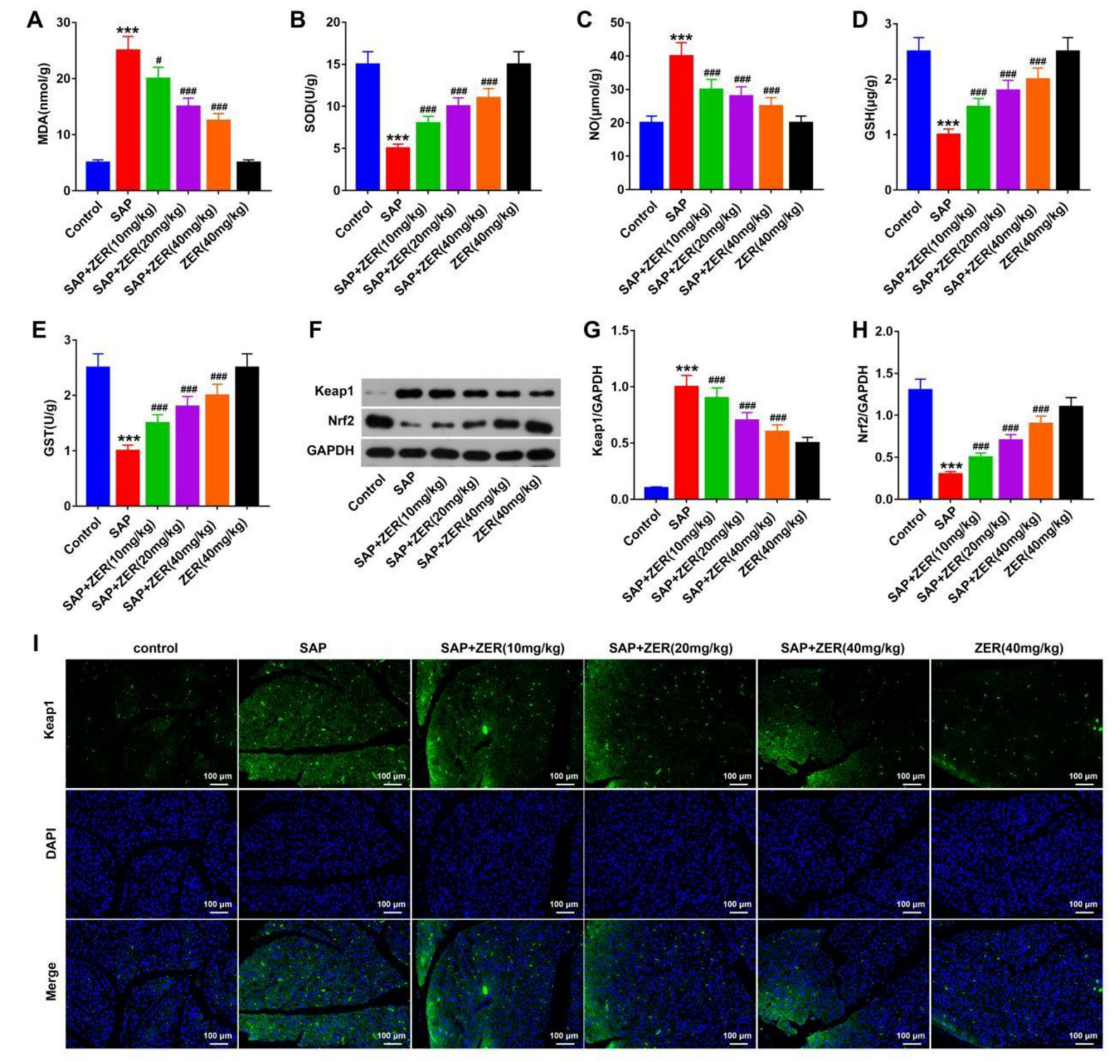
ZER attenuates oxidative stress injury in SAP-induced rats. **(A-E)** Levels of the oxidative stress factors MDA, SOD, NO, GSH and GST in each group. **(F-H)** Western blot analysis of Keap1 and Nrf2 protein expression levels. (I) Representative immunofluorescence images showing Keap1 staining in the pancreas of each group of rats (scale bar: 25 μm). ^***^P < 0.001, compared with CON group, ^#^P < 0.05, ^###^P < 0.001, compared with SAP group

Next, we evaluated the effects of ZER on oxidative stress in SAP-induced rats. SAP significantly increased MDA and NO levels, while decreasing SOD, GSH and GST levels compared to controls. However, following ZER treatment, a decrease in MDA and NO levels, and increase in SOD, GSH and GST levels were observed compared to the SAP group (p < 0.05, Fig. 2A-E). Western blot analysis revealed upregulated Keap1 and very low Nrf2 expression levels in the SAP group compared to the control group. ZER treatment led to a dose-dependent decrease in Keap1 and increase in Nrf2 expression levels compared to SAP-induced rats (P < 0.05, Fig. 2F-H). Our Keap1 immunofluorescence staining data were consistent with these results (Fig. 2I).

### ZER attenuates inflammatory damage in SAP-induced rats

**Fig. 3:**
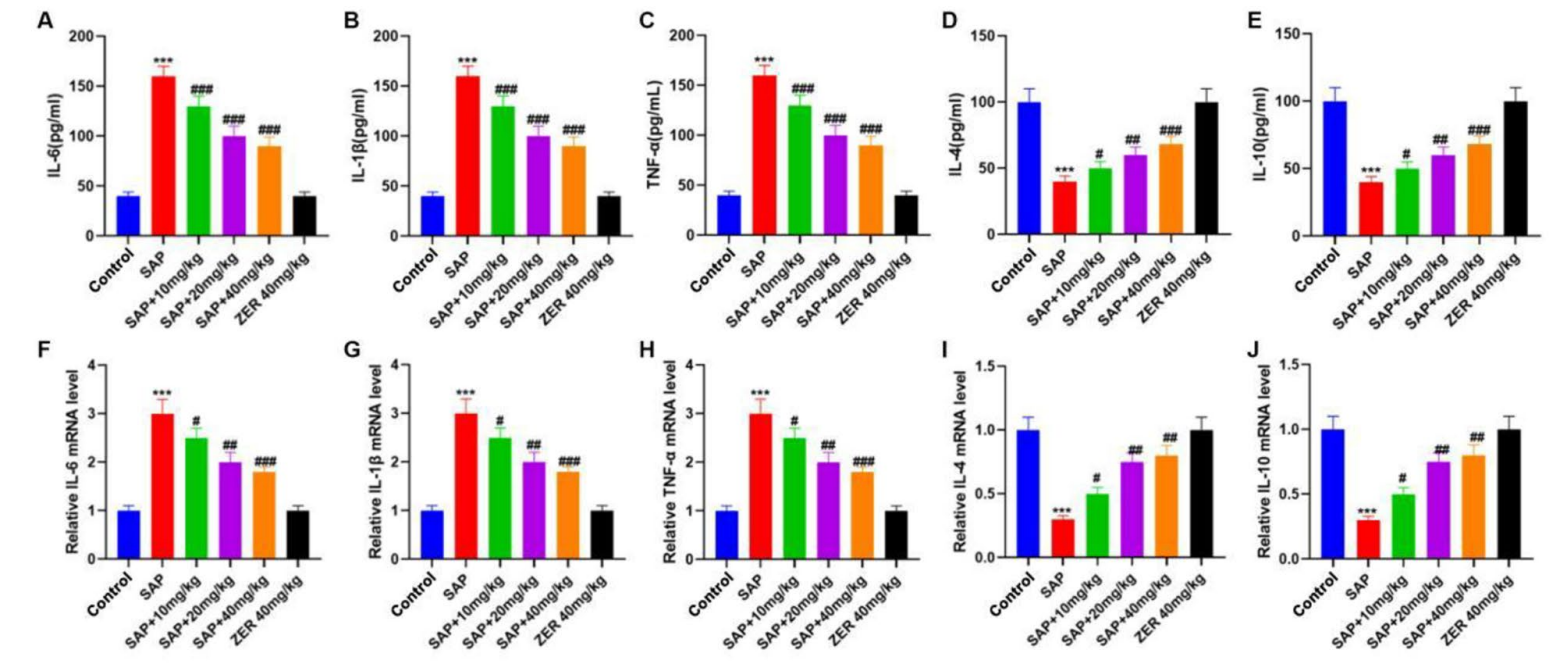
ZER attenuates inflammatory damage in SAP-induced rats. **(A-E)** ELISA was used to assess the levels of IL-6, IL-1β, TNF-α, IL-4 and IL-10. **(F-J)** The qRT-PCR was used to measure IL-6, IL-1β, TNF-α, IL-4 and IL-10 mRNA levels. ^***^P < 0.001, compared with the CON group, ^#^P < 0.05, ^##^P < 0.01, ^###^P < 0.001, compared with the SAP group

Subsequently, the effects of ZER on inflammatory damage in SAP-induced rats were evaluated. Our ELISA data revealed an increase in the levels of the inflammatory cytokines IL-6, IL-1β1 and TNF-α, together with a reduction in IL-4 and IL-10 levels in the SAP group compared to the control group. Following ZER treatment, IL-6, TNF-α and IL-1β levels were reduced and IL-4 and IL-10 levels were increased in a concentration-dependent manner compared to the SAP group (P < 0.05, Fig. 3A-E). Our qRT-PCR data were consistent with these findings (P < 0.05, Fig. 3F-J).

### ZER alleviates liver injury in rats with SAP

**Fig. 4:**
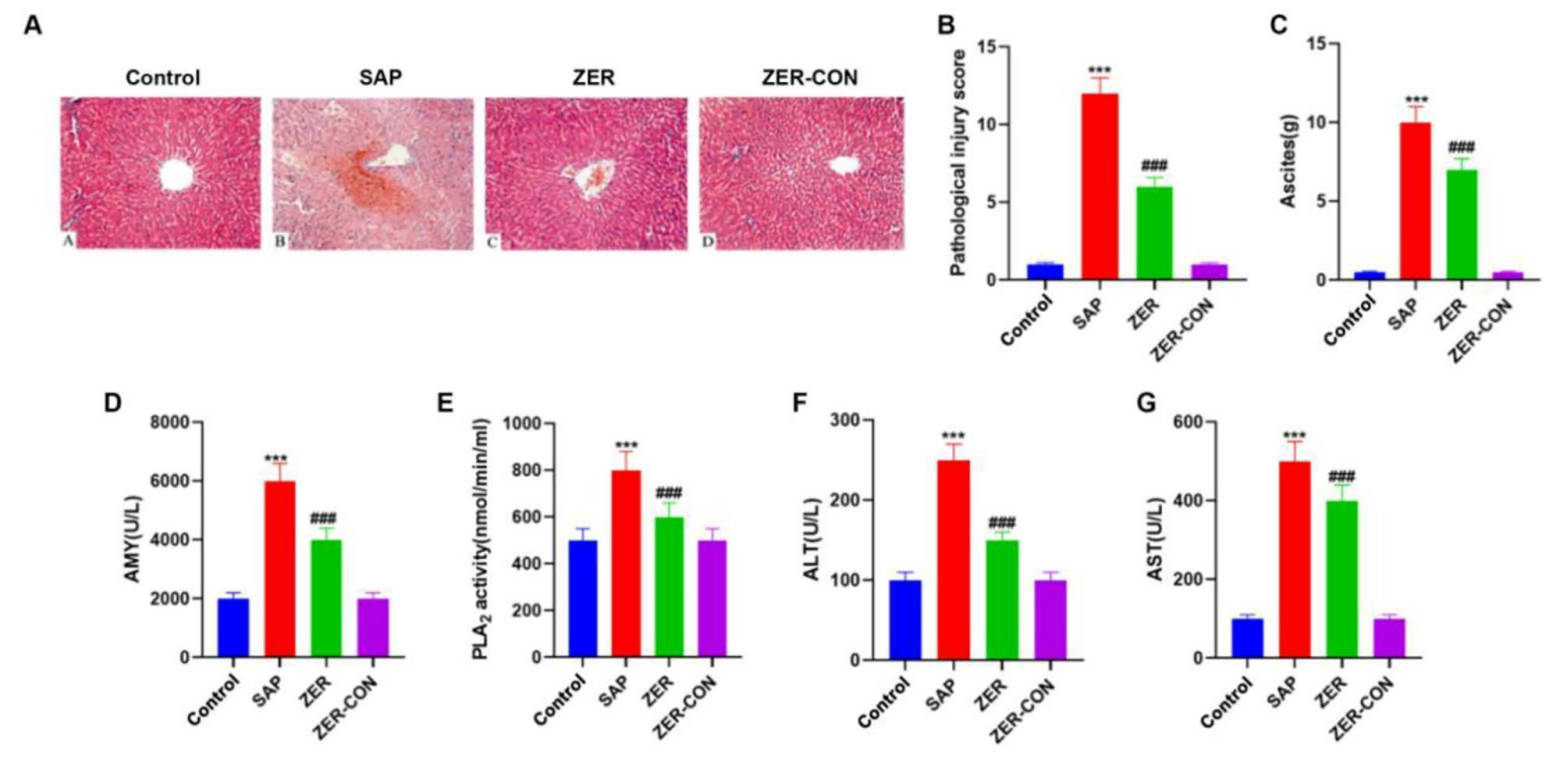
ZER alleviates liver injury in rats with SAP. **(A)** Histopathological changes in the liver of each group of rats were detected by H&E staining (×200). **(B)** Histological score of the liver pathology for each group of rats. **(C)** Measurement of abdominal water volume in each group of rats. **(D)** Measurement of serum amylase (AMY) levels in each group of rats. **(E)** Determination of serum phospholipase A_2_ (PLA_2_) levels in each group of rats. **(F)** Determination of serum alanine aminotransferase (ALT) levels in each group of rats. **(G)** Determination of aspartate aminotransferase (AST) levels in each group of rats. ^***^P < 0.001, compared with the CON group, ^###^P < 0.001, compared with the SAP group

We found that ZER (10, 20 and 40 mg/kg) was effective in relieving pancreatitis and reducing the volume of ascites, serum AMY levels and pancreatic pathology score. However, although ZER provided relief from pancreatitis at concentrations of 20 mg/kg and 40 mg/kg, these concentrations were found to be more hepatorenal toxic. Thus, rats were treated with 10 mg/kg ZER for 12 h to determine the effects of ZER on liver injury in SAP-induced rats. H&E staining revealed normal pancreatic tissue pathology in the control group with no cytoplasmic vacuole formation, and no bleeding, necrosis or small granulocyte infiltration. In the SAP group, however, a large number of cytoplasmic vacuoles were formed, the cell boundary became blurred, and hepatic necrosis, neutrophil infiltration, liver capillary congestion and bleeding were apparent. In addition, the central vein was deformed and congested, and the pathological score increased (Fig. 4A,B). After intravenous administration of 10 mg/kg ZER, a reduction in the congestion and hepatic necrosis of the liver tissue was observed compared to the SAP group. After 12 h, we found that although the morphology of the central vein had returned to normal, some congestion was still observed (Fig. 4A). However, ZER treatment led to a decrease in the pathological score (Fig. 4B). The pathological changes observed in the liver of the ZER drug control (ZER-CON) group were consistent with those in the control group (P < 0.05, Fig. 4A,B). After intravenous administration of 10 mg/kg ZER, the volume of ascites in the ZER group was markedly reduced compared to the SAP group. No significant differences were observed between the ZER-CON and control groups (P < 0.05, Fig. 4C). Next, we evaluated changes in the serum indices of rats in each treatment group. We found that serum AMY, PLA_2_, ALT and AST levels in the SAP group were significantly higher than those in the control group. Following intravenous administration of 10 mg/kg ZER, the serum indices in the ZER group were significantly lower than in the SAP group, while no significant differences were found between the ZER-CON and control groups (P < 0.05, Fig. 4D-G).

### ZER alleviates SAP through the ROS/NF-κB pathway

**Fig. 5:**
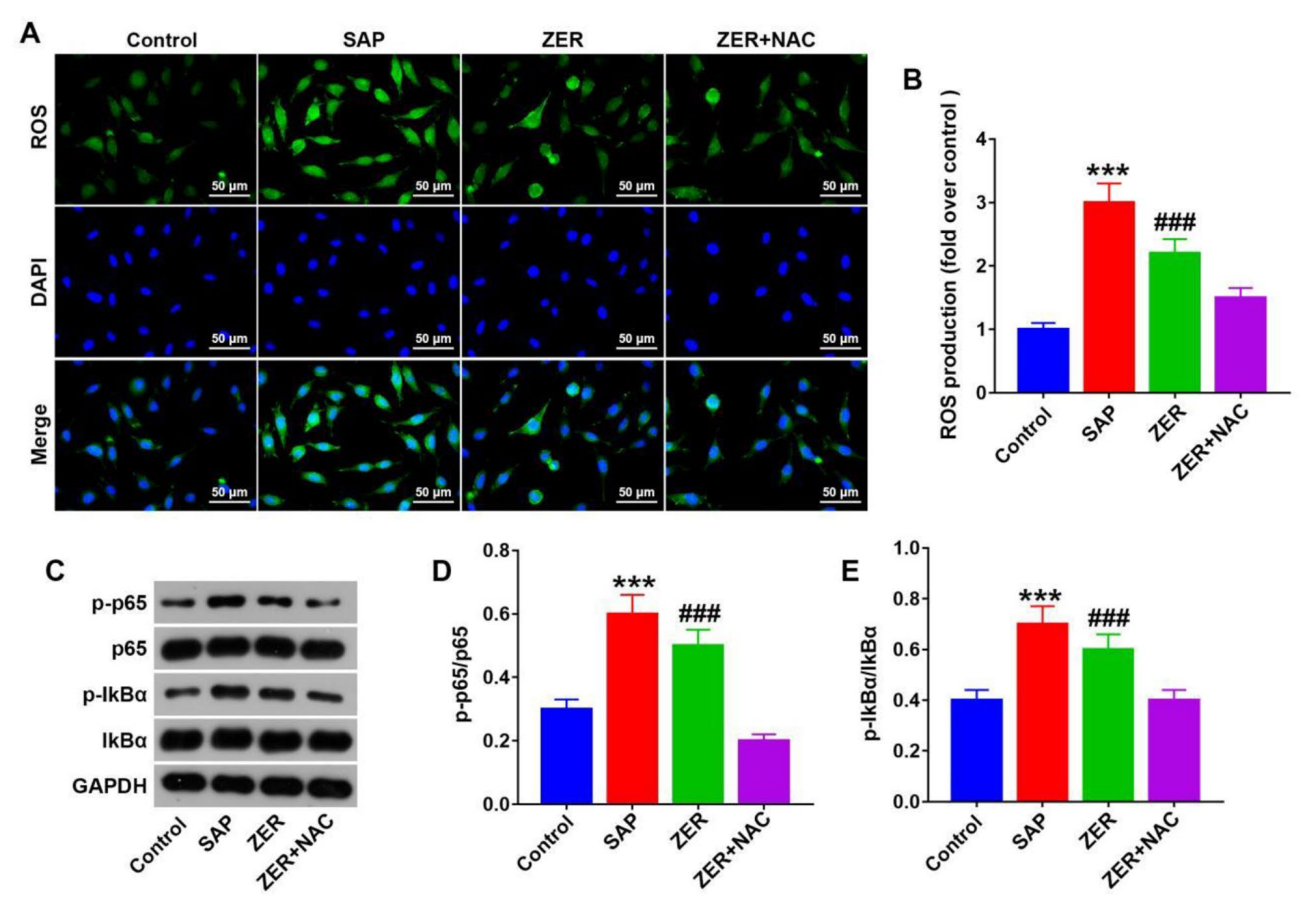
ZER alleviates SAP through the ROS/NF-κB pathway. **(A,B)** Immunofluorescence staining of ROS levels. **(C-E)** Western blot analysis was used to detect expression of NF-κB pathway-related proteins in each treatment group. Representative western blot and quantification of the western blot data are shown. ^***^P < 0.001, compared with the CON group, ^###^P < 0.001, compared with the SAP group.^@@@^P < 0.001, compared with the ZER group

Finally, we sought to determine whether the ROS/NF-κB signaling pathway had a role in mediating the effects of ZER on SAP. The ROS inhibitor NAC was injected intraperitoneally into SAP-induced rats. Our ROS staining data revealed that SAP led to an increase in relative ROS expression levels, while treatment with ZER partially reversed the effects of SAP. NAC was shown to downregulate ROS levels in SAP-induced rats (P < 0.05, Fig. 5A,B). Western blot analysis showed that SAP upregulated the relative expression levels of p65/p-p65 and I-κBα/p-I-κBα in SAP-induced rats, while ZER partially reversed the effect of SAP. NAC was found to downregulate p65/p-p65 and I-κBα/p-I-κBα levels in SAP-induced rats (P < 0.05, Fig. 5C-E).

## Discussion

Acute pancreatitis was a disease characterized by acute inflammation of the pancreas and histologic destruction of follicular cells [23]. A severe inflammatory response involving extrapancreatic organs, resulting in organ damage and failure, was indicative of progression to SAP, which had a mortality rate of 20–30% [24, 25]. Epidemiologic evidence suggested that SAP was often associated with hepatic and pulmonary injury, which may be manifested by elevated serum enzyme levels and changes in imaging, and led to a further increased risk of death [26, 27]. Currently, there was no effective treatment for SAP. Therefore, elucidating the pathogenesis of SAP was crucial to improve the overall prognosis of patients!

ZER was a multi-targeted herb with various pharmacological activities including anti-inflammatory and antioxidant properties [16, 28–30]. In the present study, the pathology of the SAP group was characterized by enlarged glandular cells, marked necrosis of the alveolar cells, and typical fat necrosis in some areas, significant inflammatory cell infiltration in the pancreatic lobules and blood vessels, which was consistent with the previous studies. Meanwhile, the lung W/D ratio in the SAP group was significantly higher than that in the control group. In addition, the levels of serum amylase, ALT, and creatinine were significantly higher in the SAP group along with the amount of ascites. All these results confirmed that the mice had developed SAP.

We compared the difference between the SAP group and the ZER groups at different doses (10, 20 and 40 mg/kg) to investigate the therapeutic effect of ZER on SAP. Our results proved that different doses of ZER were able to alleviate the progression of SAP. Specifically, the ZER groups showed a decrease in ascites volume, lower levels of amylase, ALT and Cr, and a lower pathologic score of pancreatitis. However, there was also a concentration-dependent increase in the hepatorenal toxicity of ZER. Meanwhile, the comparison of ZER control group and Control group indicated that ZER had no significant adverse effects on normal rats. Therefore, ZER at 10 mg/ kg was an ideal dose for the treatment of SAP and could serve to alleviate extra-pancreatic organ damage.

The underlying mechanism of ZER in the treatment of SAP remained unclear, and it was reported that SAP was mainly caused by oxidative stress and the release of inflammatory factors and mediators caused by pancreatic self-digestion [31–37]. Therefore, in the present study, we investigated the changes in the levels of MDA (intermediate product of oxidative stress), NO, SOD and GHS-Px (antioxidant enzymes), IL-6, IL-1β, and TNF-α [38] to investigate the effects of ZER on the levels of oxidative stress and inflammatory factors in rats with SAP. The results showed that the administration of ZER significantly decreased the levels of MDA and NO, and significantly increased the levels of GSH and GST. Meanwhile, the results showed that the levels of IL-6, IL-1β, and TNF-α decreased in a concentration-dependent manner in the ZER group, contrary to the changes in IL-4 and IL-10. Therefore, we demonstrated that ZER could attenuate oxidative stress and inflammatory factor levels in SAP mice. In addition, we verified this finding from the perspective of protein expression. Findings from Western blot showed that ZER led to a reduction in the relative expression levels of p65/p-p65 with I-κBα/I-I-κBα, which was similar to the effect of the ROS inhibitor, NAC. Thus, our results demonstrate that ZER could alleviate SAP through the ROS/NF-κB signaling pathway [39, 40].

However, this study had several limitations, and further in vivo experiments and clinical trials are needed to confirm the role of ZER in regulating ROS/NF-κB in SAP. In conclusion, this study demonstrated that ZER alleviated SAP through the ROS/NF-κB pathway. Thus, ZER may be beneficial in the treatment of SAP.

### Electronic supplementary material

Below is the link to the electronic supplementary material.

Supplementary Material 1

## Data Availability

The datasets used and/or analyzed during the current study are available from the corresponding author upon reasonable request.
