# Supplementary material for: Zerumbone ameliorates the inflammatory response and organ damage in severe acute pancreatitis via the ROS/NF-κB pathway
Source: BMC Gastroenterol. 2023 Sep 27;23:333. doi: 10.1186/s12876-023-02962-6 (PMC10538248; doi:10.1186/s12876-023-02962-6)
Supplement: Supplementary file 1 — Supplementary Material 1 [file 12876_2023_2962_MOESM1_ESM.pdf]

We are sorry we are unable to provide images showing full length membranes. Because we cut the membranes to save on antibodies when we run the experiment. We are very sorry for this and hope that this practice is allowed, thank you!

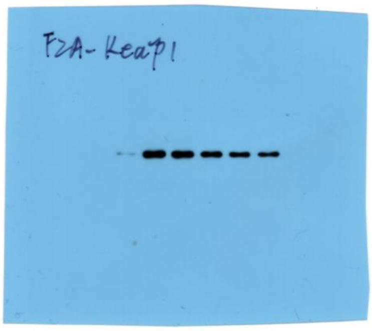

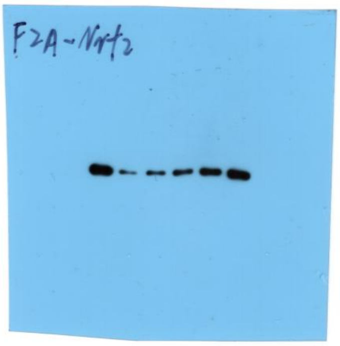

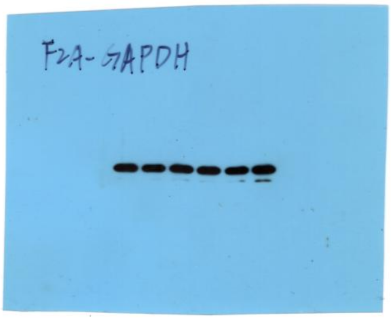

Fig2 F

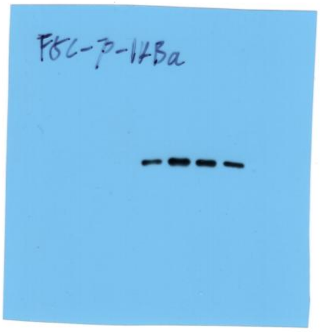
F56-p-IkBa

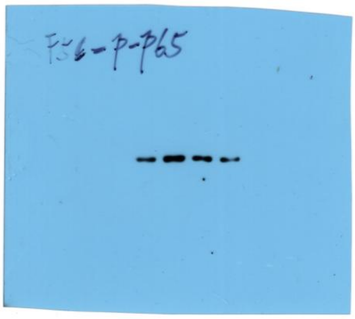
F56-p-p65

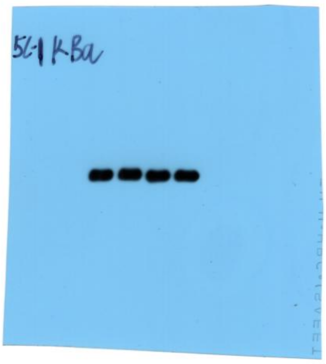
56-IkBa

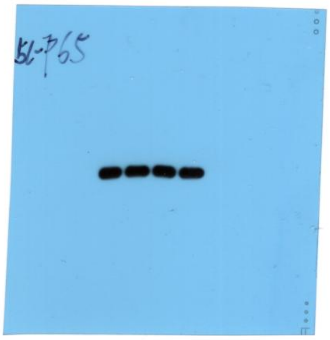
56-p65

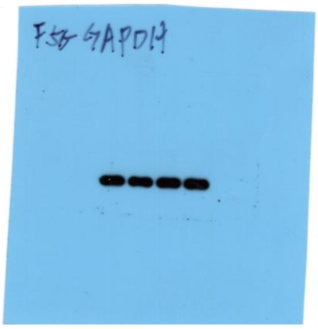
F56 GAPDH

Fig 5C
